# Vitamin D status modulates innate immune responses and metabolomic profiles following acute prolonged cycling

**DOI:** 10.1007/s00394-023-03181-1

**Published:** 2023-07-17

**Authors:** Arwel W. Jones, Adrian Mironas, Luis A. J. Mur, Manfred Beckmann, Rhys Thatcher, Glen Davison

**Affiliations:** 1 Institute of Biological, Environmental and Rural Sciences, Aberystwyth University, Aberystwyth, UK; 2 Respiratory Research@Alfred, Central Clinical School, Monash University, Melbourne, Australia; 3 School of Sport and Exercise Sciences, Division of Natural Sciences, University of Kent, Canterbury, UK

**Keywords:** 25-Hydroxyvitamin D, Neutrophil, Immunity, Inflammation, Metabolomics, Stress

## Abstract

**Purpose:**

The influence of vitamin D status on exercise-induced immune dysfunction remains unclear. The aim of this study was to investigate the effects of vitamin D status (circulating 25(OH)D) on innate immune responses and metabolomic profiles to prolonged exercise.

**Methods:**

Twenty three healthy, recreationally active males (age 25 ± 7 years; maximal oxygen uptake [${\dot{\text{V}}\text{O}}_{{2}}$max] 56 ± 9 mL·kg^−1^·min^−1^), classified as being deficient (n = 7) or non-deficient n = 16) according to plasma concentrations of 25(OH)D, completed 2.5 h of cycling at 15% Δ (~ 55–60% ${\dot{\text{V}}\text{O}}_{{2}}$max). Venous blood and unstimulated saliva samples were obtained before and after exercise.

**Results:**

Participants with deficient plasma 25(OH)D on average had lower total lymphocyte count (mean difference [95% confidence interval], 0.5 cells × 10^9^ L [0.1, 0.9]), p = 0.013) and greater neutrophil:lymphocyte ratio (1.3 cells × 10^9^ L, [0.1, 2.5], p = 0.033). The deficient group experienced reductions from pre-exercise to 1 h post-exercise (− 43% [− 70, − 15], p = 0.003) in bacterial stimulated elastase in blood neutrophils compared to non-deficient participants (1% [− 20, 21], p = 1.000) Multivariate analyses of plasma metabolomic profiles showed a clear separation of participants according to vitamin D status. Prominent sources of variation between groups were purine/pyrimidine catabolites, inflammatory markers (linoleic acid pathway), lactate and tyrosine/adrenaline.

**Conclusion:**

These findings provide evidence of the influence of vitamin D status on exercise-induced changes in parameters of innate immune defence and metabolomic signatures such as markers of inflammation and metabolic stress.

**Supplementary Information:**

The online version contains supplementary material available at 10.1007/s00394-023-03181-1.

## Introduction

The identification of the vitamin D receptor (VDR) and vitamin D metabolising enzymes in immune cells such as monocytes, macrophages and neutrophils has led to recognition of the important role that vitamin D has in the regulation of immune responses [1]. Vitamin D is essential in the interplay between recognition of microbes and the activation of antimicrobial responses in innate immunity [2]. VDR genes are in close proximity to genes that encode antimicrobial peptides (AMPs) [3] with vitamin D in its active form (1,25 dihydroxy cholecalciferol) up-regulating the expression of AMPs such as cathelicidin and β-defensin [4, 5]. Vitamin D metabolites can directly enhance key effector functions (chemotaxis, phagocytosis and generation of reactive oxygen species) of monocytes, macrophages and neutrophils [6–8].

It is well accepted that transient perturbations of cellular and mucosal immunity occurs following prolonged, strenuous exercise (commonly referred to as an ‘open window’ of immunodepression) [9]. If such exercise is performed in combination with other life stressors (e.g. inadequate nutrition, psychological stress) there is likely to be an increased susceptibility to upper respiratory illness (URI) [10]. One factor purported to predispose individuals to more frequent URI is vitamin D status [11]. Low vitamin D status compared to greater circulating vitamin D concentrations has been associated with greater incidence of URI in endurance athletes (67% vs 27%) or military personnel undergoing training (9% vs 6%) [2, 12]. Lower vitamin D status is commonly reported within athletes at higher latitudes, in winter and early spring seasons and/or as a result of minimal sun exposure (e.g. involvement in indoor sport activities, [13]). Despite recognition of the potential for variations in vitamin D status to have direct effects on immunity [14], the influence of circulating concentrations of vitamin D on acute bouts of exercise-induced immune dysfunction remains unclear.

Circulating concentration of 25(OH)D is considered to be a primary indicator of vitamin D status. There is, however, a longstanding debate to what concentrations of 25(OH)D constitute vitamin D deficiency, insufficiency, sufficiency and toxicity [15]. Given that vitamin D is linked to a host of biological effects, it is plausible to suggest that the optimal level may depend on the specific physiological function or health outcome. There is currently no consensus on the optimal thresholds of 25(OH)D for the immune system [16]. Of the limited immunological studies in athletic populations, lower vitamin D concentrations (< 33 nmol/L) has been reported to be associated with lower production of pro-inflammatory cytokines by innate phagocytes (monocytes) in response to a multi-antigen challenge in athletes and lower plasma cathelicidin concentrations [2]. In a study of military personnel immune response to hepatitis B vaccine was poorer in those with circulating 25(OH)D of ≤ 40 nmol/L (mean 30 ± 7 nmol/L) compared to participants with 25(OH)D between 41 and 71 nmol/L (mean 56 ± 9 nmol/L) at the time of initial vaccination. Some [17] but not all intervention studies [12] in these populations have demonstrated that markers of innate immunity (plasma cathelicidin, saliva flow rates) are amenable to change with oral vitamin D supplementation (daily capsule doses of 400–5000 IU for 12–14 weeks).

Despite such findings of vitamin D status (~ < 33 nmol/L) markers of innate immunity at rest, further human mechanistic research that adopts systems biology approaches is warranted to clarify whether the vitamin D status is having physiological effects or simply correlated with such events [18, 19] (i.e. marker of immunodepression). Strenuous exercise has a profound effect on human metabolism albeit most studies have focused on a narrow range of metabolites [20]. Metabolomics conveys an innovative approach for phenotyping individuals at a molecular level, offering the possibility to simultaneously and accurately measure hundreds of metabolites [21]. It allows for unbiased knowledge expansion of many metabolic pathways and complex interactions within the body during exercise or nutrition interventions [22, 23]. Although vitamin D status has been shown to influence metabolic profiles [24, 25], to date there have been a lack of published studies illustrating the effects of vitamin D status on metabolic modifications following prolonged exercise. Furthermore, there remains a lack of investigations in exercise immunology research that use high throughput laboratory methods such as metabolomics alongside markers of immunity to provide a greater understanding of the mechanisms behind any modulatory effects of exercise and/or nutrition.

The primary aim of our study was to investigate the effects of vitamin D status on innate immune responses to prolonged exercise. Secondly, we undertook a metabolomic profiling approach to suggest how the immune system may be modulated in relation to plasma 25(OH)D concentrations.

## Materials and methods

### Design

This is a secondary analysis of two randomised controlled trials of nutritional interventions (acute and 4 weeks of bovine colostrum supplementation) and immune responses to prolonged exercise [26]. The participants in this study took part in the control (placebo) arm of these trials.

### Participants

Twenty three healthy, recreationally active males (age 25 ± 7 years; 96% white ethnicity; body mass [BM] 76 ± 8 kg; height 179 ± 6 cm; maximal oxygen uptake [${\dot{\text{V}}\text{O}}_{2}$max] 56 ± 9 mL·kg^−1^·min^−1^) volunteered to participate. The study was conducted in accordance with the Declaration of Helsinki principles. Aberystwyth University Research Ethics Committee approved all experimental procedures prior to the recruitment of any participants. Participants provided both verbal and written consent following information on experimental procedures. All participants were non-smokers and reported no symptoms of infection or consumption of any medication or dietary supplements for 4 weeks prior to commencement of, and during, the study.

### Preliminary testing

Fourteen days prior to the main experimental visit, gas exchange threshold (GET) and ${\dot{\text{V}}\text{O}}_{2}$max were determined via a continuous incremental test (30 W∙min^−1^ ramp rate following 3 min of unloaded baseline pedalling) to volitional exhaustion on an electrically braked cycle ergometer (Lode Excalibur, Groningen, The Netherlands). Throughout the duration of the incremental test, expired gas was analysed by the use of an online breath-by-breath gas analysis system (Jaeger Oxycon Pro, Hoechberg, Germany). The test was terminated when the participant’s cadence fell 10 rpm below their preferred cadence for more than 10 s as described previously [27]. For each participant, ${\dot{\text{V}}\text{O}}_{2}$max was determined by the highest 30 s average during the test. GET was estimated for each participant via the V-slope method [28]. The exercise intensity was set to the power output that would elicit 15% Δ (15% of the difference between power output at GET and ${\dot{\text{V}}\text{O}}_{2}$max) which was equivalent to ~ 55–60% of ${\dot{\text{V}}\text{O}}_{2}$max in these participants. The use of % Δ was used to provide more accurate control of the relative intensity and better standardize inter-subject physiological demand and responses [27]. Seven days prior to the main experimental visit, a familiarisation trial took place to accustom participants to the testing procedures and physical stress expected in the main experimental trial. Participants performed a 2.5 h exercise bout on the electronically braked cycle ergometer (specified above) at an intensity of 15% Δ. Expired gas was analysed during the 10th, 30th, 60th, 90th and 120th min of exercise to verify that the selected workload did elicit the target intensity. Heart rate (HR) and RPE were monitored every 15 min during the protocol using a telemetric device (Polar S610, Polar Electro Oy, Kempele, Finland) and Borg scale respectively [29].

### Experimental trial procedures

During the 48 h preceding the experimental trial, participants were asked to refrain from heavy exercise and alcohol consumption. On the morning of the experimental trial, participants reported to the laboratory at 09:00 after an overnight fast of at least 10 h. The participants were asked to consume 500 mL of water 2 h before arrival to encourage euhydration. Participants remained seated for 10 min prior to collection of a resting blood sample from an antecubital vein and an unstimulated saliva sample (see details on sampling below). Following collection of samples, participants immediately commenced 2.5 h of cycling at 15% Δ. All participants were permitted diluted cordial (four volumes of water to 1 volume of sugar-free cordial at 2 mL· kg of BM) every 15 min during the exercise but not at end of the exercise (to limit contamination to saliva samples). Expired gas was analysed during the 30th, 60th, 90th and 120th min of exercise (Jaeger Oxycon Pro, Hoechberg, Germany). HR and RPE were monitored every 15 min during the protocol. Participants remained fasted for further blood and saliva samples immediately and 1 h post-exercise.

### Blood sampling

Participants remained seated, performing minimal movement for 10 min prior to each blood sample with the exception of immediately post-exercise samples that were drawn within a few min of exercise cessation. Blood was collected pre-exercise, post-exercise and 1 h post-exercise. Blood samples were collected by venepuncture (with a 21 gauge PrecisionGlide needle [Becton–Dickinson, Oxford, UK]) from an antecubital vein into vacutainers (Becton–Dickinson, Oxford, UK) containing tripotassium ethylene diamine tetraacetic acid (K_3_EDTA) or lithium heparin. Haemoglobin, total and differential leukocyte counts were measured in each K_3_EDTA vacutainer using an automated haematology analyser (Pentra 60 C + Haematology analyser, HORIBA Medical, Montpellier, France). Haematocrit was determined from an aliquot of whole blood (heparin anti-coagulated) by a standard microcentrifugation method (using a Hawksley microcentrifuge). This was used along with the previously attained haemoglobin concentration, to estimate changes in blood and plasma volume from pre- to post-exercise as previously described [30]. The remaining blood in heparin and K_3_EDTA vacutainers were centrifuged at 1500 *g* for 10 min at 4 °C with subsequent plasma being stored at − 80 °C for later analysis of Vitamin D status (25(OH)D), elastase and metabolomics profiling.

### In vitro blood neutrophil function

Whole blood from the K_3_EDTA treated tubes at pre-exercise, post-exercise and 1 h post-exercise was placed in a microcentrifuge tube and stored at room temperature (no longer than 2 h) prior to measurement of in vitro stimulated neutrophil oxidative burst response to PMA using a commercially available chemiluminescence (CL) kit (ABEL, Knight Scientific Ltd, Plymouth, UK) in accordance with previous studies [27, 31]. The CL per well was measured by a microplate luminometer (FLUOstar OPTIMA, BMG Labtech, Aylesbury, UK).

In all PMA-stimulated samples, CL was recorded in duplicate as relative light units (RLU) at 20 s intervals for 30 min and the area under the CL curve was calculated. The area under the unstimulated CL curve for each sample was subtracted from the mean area of the duplicate stimulated sample to determine the PMA-stimulated CL. To account for oxidative burst responses on a per cell basis, it was assumed that the CL responses were attributable largely to the neutrophils within the samples [32]. Thus, PMA-stimulated area under the CL curve was divided by the number of neutrophils present in each well to give CL in RLU (i.e. oxidative burst response) per neutrophil.

The neutrophil degranulation response was assessed in accordance with previous research [27, 33]. The measurement of neutrophil degranulation involved adding 1 mL of the heparinised blood sample to microcentrifuge tubes containing 50 μL of stimulant containing extracts of *Staphylococcus aureus, Pseudomonas fluorescens* and *Enterobacter aerogenes* (840–15, Sigma, Poole, UK). The tubes were initially mixed by gentle inversion before being incubated at 37 °C for 1 h. All tubes were gently mixed halfway through the incubation period. Following incubation, the tubes were centrifuged for 2 min at 16,000 *g*, with the supernatant being immediately removed and stored at − 80 °C until further analysis. Upon thawing at room temperature, neutrophil degranulation response was based on measuring the amount of stimulated elastase release per neutrophil using an ELISA kit (Merck Calibiochem, Darmstadt, Germany). Bacterial-stimulated elastase release was calculated as the difference between stimulated and unstimulated samples elastase concentration. The unstimulated samples were processed immediately to provide background plasma elastase concentration at the specific timepoint (i.e. not incubated alongside stimulated samples).

To facilitate inter-subject comparisons, post-exercise and 1 h post-exercise neutrophil responses were expressed as a percentage of the pre-exercise value, in accordance with previous studies [27, 34].

### Plasma 25(OH)D

EDTA plasma samples were thawed to room temperature and deproteinised prior to analysis of Total 25(OH)D (25(OH)D_3_ and 25(OH)D_2_) in an independent laboratory (Department of Clinical Biochemistry, Prince Charles Hospital, Merthyr Tydfil, UK). Following precipitation of samples, 25(OH)D (25(OH)D_3_ and 25(OH)D_2_ were measured in accordance with the Chromsystems 25 OH Vitamin D liquid chomotatography-tandem mass spectrometer kit and via use of a liquid chomotatography-tandem mass spectrometer (Agilent 6410) operating in multiple reactions monitoring mode. Intra assay coefficient of variation was < 10%.

### Plasma metabolomics

Metabolites were extracted following the procedure described by Jones et al. [21]. Glass vials were capped and analysed in random order on a LTQ linear ion trap (Thermo Electron Corporation). Data were acquired in alternating positive and negative ionization modes over 4 scan ranges (15–110, 100–220, 210–510, and 500–1200 *m/z*), with an acquisition time of five minutes. Discriminatory metabolites were selected and tentatively identified by following statistical analyses and interrogation of the KEGG (Kyoto Encyclopedia of Genes and Genomes) (http://www.kegg.jp/) and human metabolome (http://www.hmdb.ca/) databases. To substantiate these identifications, nominal mass signals were investigated further by targeted nano-flow Fourier Transform-Ion Cyclotron Resonance Ultra-Mass-Spectrometry (FT-ICR-MS) using TriVersa NanoMate (Advion BioSciences Ltd) on a LTQ-FT-ULTRA (Thermo Scientific) to obtain ultra-high accurate mass information and MSn ion-trees. Based on an accuracy of 1 ppm for the FT-ICR-MS, the top ranking metabolite with this range was indicated as the identification for each discriminatory negative ionisation mode metabolite.

The concentrations of linoleic and arachidonic acid in the MS spectra were calculated based on the derivation of a standard curve (0.001, 0.01, 0.1 and 1 mg/mL) based on percentage of total MS ion score.

### Saliva sampling

Participants remained seated, performing minimal movement for 10 min prior to each saliva sample with the exception of immediately post-exercise samples, which were obtained within a few minutes of exercise cessation. Saliva samples were collected following blood samples at pre-exercise, post-exercise and 1 h post-exercise. For all saliva samples the mouth was rinsed with plain water at least 10 min before the collection period. The participant was requested to swallow in order to empty the mouth before each saliva sample. To obtain the sample, the participant remained seated with the head tilted slightly forward and passively dribbling into a pre weighed 7 mL sterile bijou tube while keeping orofacial movement to a minimum. The final duration of collection was recorded and the tube was re-weighed to allow for calculation of saliva flow rate when the density of saliva was assumed to be 1.0 g·ml^−1^ as used previously [33].

After collection of saliva, samples were centrifuged for 5 min at 16,000 *g* to pellet debris leaving the remaining clear supernatant to be aliquoted and stored at – 80 °C for later analysis. All saliva samples were thawed at room temperature only once prior to analysis. Following the thawing of saliva, samples were again centrifuged for 5 min at 16,000* g* to precipitate mucins and other debris and allow for the resulting clear supernatant to be analysed. With the use of a freezing point depression osmometer (Osmomat 030, Gonotec, GmbH, Berlin, Germany), saliva osmolality was determined to allow for concentration of salivary immunological parameters to be expressed relative to saliva osmolality.

Aliquots of saliva were screened for blood contamination by the determination of salivary transferrin concentration using an ELISA kit (Salivary blood contamination enzyme immunoassay kit, Salimetrics, State College, Pennsylvania, USA). If salivary transferrin concentration was greater than 1 mg dL^−1^, the sample was considered contaminated with blood and all other salivary data for that participant were excluded.

### Salivary antimicrobial peptides

The concentrations of antimicrobial peptides were determined in accordance with the methods of Jones et al. [21]. Following a 1000 and 8000 fold dilution of saliva supernatants (with PBS) commercially available ELISA kits were used to measure the concentration of salivary lactoferrin (sLac) and lysozyme (sLys respectively (Assaypro LLC, St-Louis, MO).

### Statistical analysis

A formal sample size calculation was not performed for the current study. The sample size was determined by the available participant data from the control arms of two previous randomised controlled trials. Data shown in the text, tables and figures are presented as mean ± standard deviation unless stated otherwise. Statistical analysis of all data was performed via the statistical computer software package SPSS (v25.00; SPSS Inc., Chicago, IL, USA). Initially, a two factor mixed ANOVA (group × time) was carried out on immunological measures to determine if the effect of time was different among groups classified by Vitamin D status. Based on data on plasma 25 (OH)D and innate immune responses (plasma cathelicidin, monocyte-derived cytokine production) in endurance athletes [2], participants were stratified to groups with deficient (< 33 nmol/ L) or non-deficient (> 33 nmol/L) plasma total 25(OH)D. Any significant main effects of group or group × time interaction identified in the ANOVA were further analysed by post hoc *t* tests with Holm–Bonferoni correction (between groups at specific time points) and one way repeated measures ANOVA within each group (also followed by post hoc *t* tests with Holm–Bonferoni correction, between times within group, where necessary). Correlations between post-exercise neutrophil responses and the plasma 25(OH)D concentration were also carried out using Spearman's rank correlation coefficient. Independent *t* tests were used to determine any significant differences between groups in HR, oxygen uptake $\left( {{\dot{\text{V}}\text{O}}_{2} } \right)$ or RPE during the main experimental trial. Statistical significance was accepted at p < 0.05. FIE-MS data were normalised with the total ion count for each sample used to transform the intensity value for each metabolite in to a percentage of the total ion count, after the removal of metabolites below 50 *m/z*. Multivariate analyses of metabolomic datasets (Principal Component Analyses [PCA] and ANOVA, Hierarchical Cluster Analyses (HCA) were completed using the R-based MetaboAnalyst 2.0 interface [35].

## Results

### Baseline characteristics, physiological responses and RPE

Baseline characteristics of the study participants are provided in Table 1. There was no significant difference in during the main experimental trial when expressed in either absolute terms (p = 0.224) or relative to ${\dot{\text{V}}\text{O}}_{{2}}$max (p = 0.549) between the groups (deficient: 2185 ± 252 mL·min^−1^, 55 ± 2% ${\dot{\text{V}}\text{O}}_{{2}}$max; non-deficient: 2458 ± 544 mL·min^−1^; 56 ± 6% ${\dot{\text{V}}\text{O}}_{{2}}$max). There was no difference (p = 0.994) in HR responses to the experimental trial between participants with plasma 25(OH)D concentration of deficient (134 ± 18 bpm) and non-deficient (134 ± 11 bpm) groups. There was also no significant difference in RPE (p = 0.311) between the two groups (deficient: 12.5 ± 0.7; > non-deficient: 13.0 ± 1.2). There was no effect of exercise or any differences present between groups in blood glucose concentration (two-way ANOVA, group, p = 0.640; time, p = 0.385; group × time interaction, p = 0·612). Similar patterns of plasma volume changes were observed from pre-exercise between trials: deficient; post-exercise (− 2.5 ± 4.2%); 1 h post-exercise (0.79 ± 4.9%) and non-deficient; post-exercise (− 4.4 ± 2.7%), 1 h post-exercise (− 1.3 ± 4.8%). As there was no significant difference between trials (group ˣ time interaction; p = 0.549), it was deemed unnecessary to correct any haematological parameters for plasma volume changes.

**Table 1:** Baseline characteristics

Group	Deficient	Non-deficient	p
Age, yrs	25 ± 4	25 ± 8	0.948
Body mass, kg	77 ± 8	75 ± 8	0.560
Height, cm	181 ± 7	179.0 ± 5	0.374
${\dot{\text{V}}\text{O}}_{2}$ max mL·kg^−1^·min^−1^	55 ± 11	56 ± 8	0.758

### Circulating total and differential cell counts

Analysis revealed a significant main effect of group on total lymphocyte count (p = 0.013) and neutrophil:lymphocyte ratio (p = 0.033). The group with deficient vitamin D status had an overall lower lymphocyte count and greater neutrophil:lymphocyte ratio compared to their counterparts with non-deficient vitamin D concentrations. There was no time × group interaction effect or main effect of group on circulating total leukocytes, neutrophils, and monocytes (Table 2). A main effect of time (p < 0.001) was observed in all leukocyte counts (Table 2). There was a significant increase in total leukocytes, neutrophils, monocytes, neutrophil:lymphocyte ratio from pre-exercise to post-exercise (p ≤ 0.01) and 1 h post exercise (except lymphocytes) (p < 0.01). There was a significant increase in, neutrophil:lymphocyte ratio (p < 0.001) and decrease in total lymphocytes (p < 0.001) and monocytes (p = 0.017) from post-exercise to 1 h post-exercise.

**Table 2:** Blood immune cell counts in Vitamin D groups

Cell count, 10^9^ L^−1^	Pre-exercise	Post-exercise	1 h post-exercise	p values group; time; interaction
Total leukocytes				0.368; < 0.001*; 0.137
Deficient	5.54 ± 1.56	10.26 ± 2.26	10.56 ± 1.11	
Non-deficient	5.15 ± 0.93	12.24 ± 3.50	11.55 ± 3.10	
Neutrophils				0.695; < 0.001*; 0.164
Deficient	3.51 ± 1.65	7.59 ± 2.33	8.34 ± 0.90	
Non-deficient	2.75 ± 0.62	8.76 ± 2.80	8.84 ± 2.67	
Monocytes				0.771; < 0.001*;0.472
Deficient	0.48 ± 0.15	0.76 ± 0.24	0.72 ± 0.19	
Non-deficient	0.48 ± 0.11	0.84 ± 0.28	0.72 ± 0.20	
Total lymphocytes				0.013†; 0.001*; 0.287
Deficient	1.34 ± 0.31	1.69 ± 0.55	1.29 ± 0.27	
Non-deficient	1.71 ± 0.44	2.33 ± 0.61	1.74 ± 0.37	
Neutrophil:lymphocyte				0.033†; < 0.001*; 0.799
Deficient	2.85 ± 1.79	5.09 ± 2.67	6.66 ± 1.36	
Non-deficient	1.69 ± 0.48	3.87 ± 1.22	5.15 ± 1.48	

†Signifcant main effect of group; *Signifcant main effect of time

### Neutrophil responses

**Fig.1:**
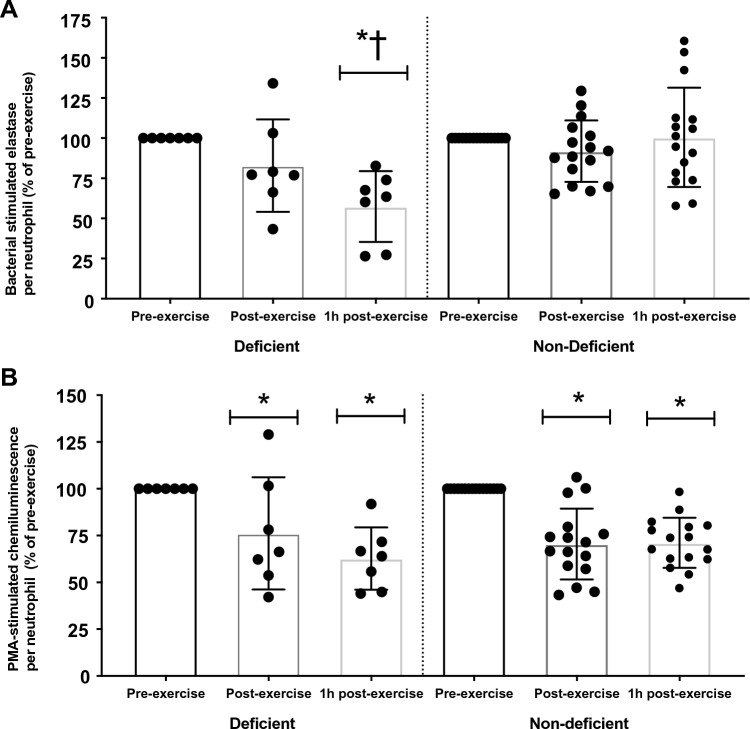
Bacterial stimulated elastase release (**A**) and PMA stimulated chemiluminescence (**B**) per neutrophil following prolonged exercise according to Vitamin D status. *Significantly different to pre-exercise (p < 0.05); †Significantly different to 1 h post-exercise in non-deficient (p < 0.05)

Two-way mixed ANOVA revealed a significant main effect of group (p = 0.010) for stimulated elastase release per neutrophil (neutrophil degranulation) with lower responses in the deficient group. A statistically significant group × time interaction (p = 0.003) was also observed for stimulated elastase release per neutrophil (neutrophil degranulation) (Fig. 1A). One-way ANOVA on each group revealed a significant main effect of time in the deficient group (p = 0.006) but not in the non-deficient (p = 0.383). Further post hoc analysis of the deficient group revealed a significant decrease from pre-exercise to 1 h post-exercise (p = 0.007). Two-tailed independent *t* tests between groups at each timepoint also revealed significantly lower bacterial stimulated elastase concentration in the deficient group at 1 h post-exercise (p = 0.003). Furthermore, across all participants there was a positive correlation between the plasma 25(OH)D and change in bacterial stimulated elastase concentration from pre-exercise to 1 h post exercise (r = 0.458, P = 0.028).

A significant main effect of time (p < 0.001) was observed for PMA-stimulated CL per neutrophil (neutrophil oxidative burst) (Fig. 1B). There was a significantly lower PMA-stimulated CL per neutrophil at post-exercise and 1 h post-exercise compared with pre-exercise (p = 0.001). There was no main effect of group (p = 0.857) or group ×time interaction (p = 0.258) for PMA-stimulated CL per neutrophil (Fig. 1B).

### Plasma metabolomics

**Fig. 2:**
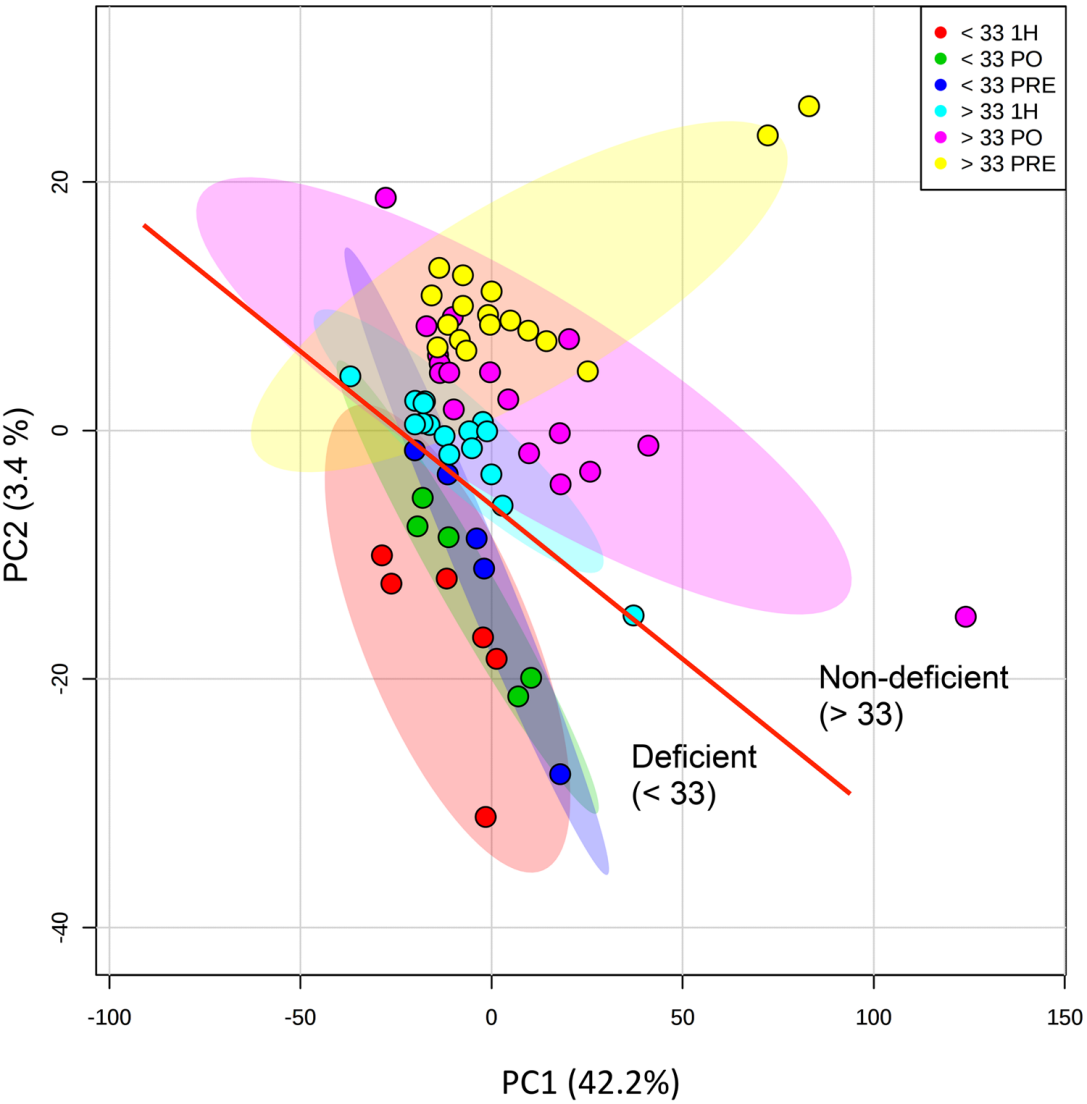
Differentiation of the plasma metabolomes according to exercise and vitamin D status. Principal component analysis of plasma metabolite profiles from participants sampled pre-exercise (PRE), immediately post-exercise (PO) and 1 h post-exercise (1H). Analyses were also subdivided into participants with deficient (< 33 nmol/L) or non-deficient (> 33 nmol/L) plasma vitamin D concentrations. The coloured circles indicate 95% confidence intervals for each experimental class. The red line and the indications of groups < 33 or > 33 nmol/L vitamin D are for illustrative purposes and have no mathematical value

A metabolomics approach was used to determine the effect of vitamin D status on metabolic responses to prolonged exercise. Plasma metabolite profiles at each of the three time points were screened using mass spectrometry and subsequently analyzed using multivariate approaches. Application of PCA showed a separation between the two samples sets (vitamin D status groups) and some differences in responses to exercise (Fig. 2). Thus, separation was observed between post-exercise and 1 h post-exercise samples in participants with deficient plasma (25OH)D concentration.

**Fig. 3:**
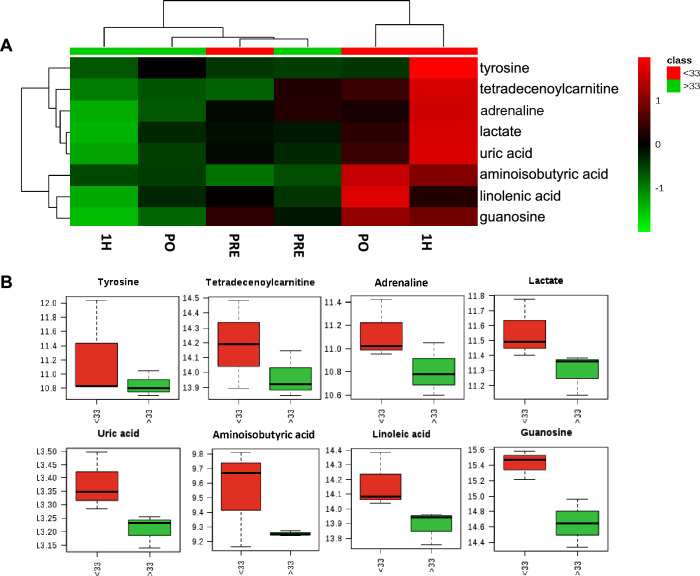
Major metabolite changes in plasma pre- and post-exercise in participants with different vitamin D status. ANOVA identified the metabolites that were the major source of variation in the metabolomes of participants sampled pre-exercise (PRE), immediately post-exercise (PO) and 1 h post-exercise (1H). Analyses were also subdivided into participants with deficient (< 33 nmol/L) or non-deficient (> 33 nmol/L) plasma vitamin D concentrations. (A) mean values for each metabolite and category are indicated using a heat map and hierarchical cluster analyses. (B) Variation in metabolites for deficient (< 33 nmol/L) and non deficient (> 33 nmol/L) classifications are indicated using box and whisker plots

Two factor mixed ANOVA (group × time), corrected for false discovery rate, identified *m/z* that were calculated to be the major sources of variation. Interrogation of the KEGG and human metabolome database allowed putative identification of the corresponding metabolites (Table 3). The samples were assessed with high resolution mass spectrometry with a *m/z* accuracy of 1 ppm to further confirm the identity of the metabolites. The mean values for each sample group were displayed using a heat map and dendrogram (Fig. 3A) to indicate that the metabolites in the non-deficient group did not greatly change during the exercise period. The levels of these metabolites did not differ in the pre-exercise deficient samples. However, following exercise, and particularly 1 h post-exercise, all metabolites were significantly elevated only in the deficient group. These post-exercise increases in the deficient group were also shown when results were plotted using box and whisker graphs (Fig. 3B).

**Table 3:** Major sources of variation in the effect of Vitamin D status on metabolomic responses to prolonged exercise

Nominal mass	Tentative ID	F-value	P-value	KEGG/HDMI pathway	Comments
103.0633	Aminoisobutyric acid	5.16	0.001	map00240	Pyrimidine metabolism
280.2402	Linoleic acid	4.908	0.001	map01040	Biosynthesis of unsaturated fatty acids
283.0917	Guanosine	2.856	0.024	map00230	Purine metabolism
181.0739	Tyrosine	2.793	0.026	map00350	Catecholamine biosynthesis, tyrosine = > dopamine = > noradrenaline = > adrenaline
369.5396	Tetradecenoylcarnitine	2.578	0.037	MDB02014	Tetradecenoylcarnitine (C14:1) is the most characteristic metabolic marker of very long chain acyl-dehydrogenase (VLCAD) deficiency
168.1106	Uric acid	2.463	0.045	map00230	Purine metabolism
90.0781	Lactate	2.011	0.056	map00620	Pyruvate metabolism
183.0898	Adrenaline	2.093	0.059	map00350	Tyrosine metabolism

Considering the biochemical function of the metabolites (Table 3), increases in lactate suggested increased anaerobic respiration in the deficient group. Physical exertion is accompanied by increased degradation of  nucleotides in muscles and purine/pyridine degradation products were increased with low vitamin D. Increases in tyrosine/adrenaline were consistent with elevated stress during exercise in the deficient group.

**Fig. 4:**
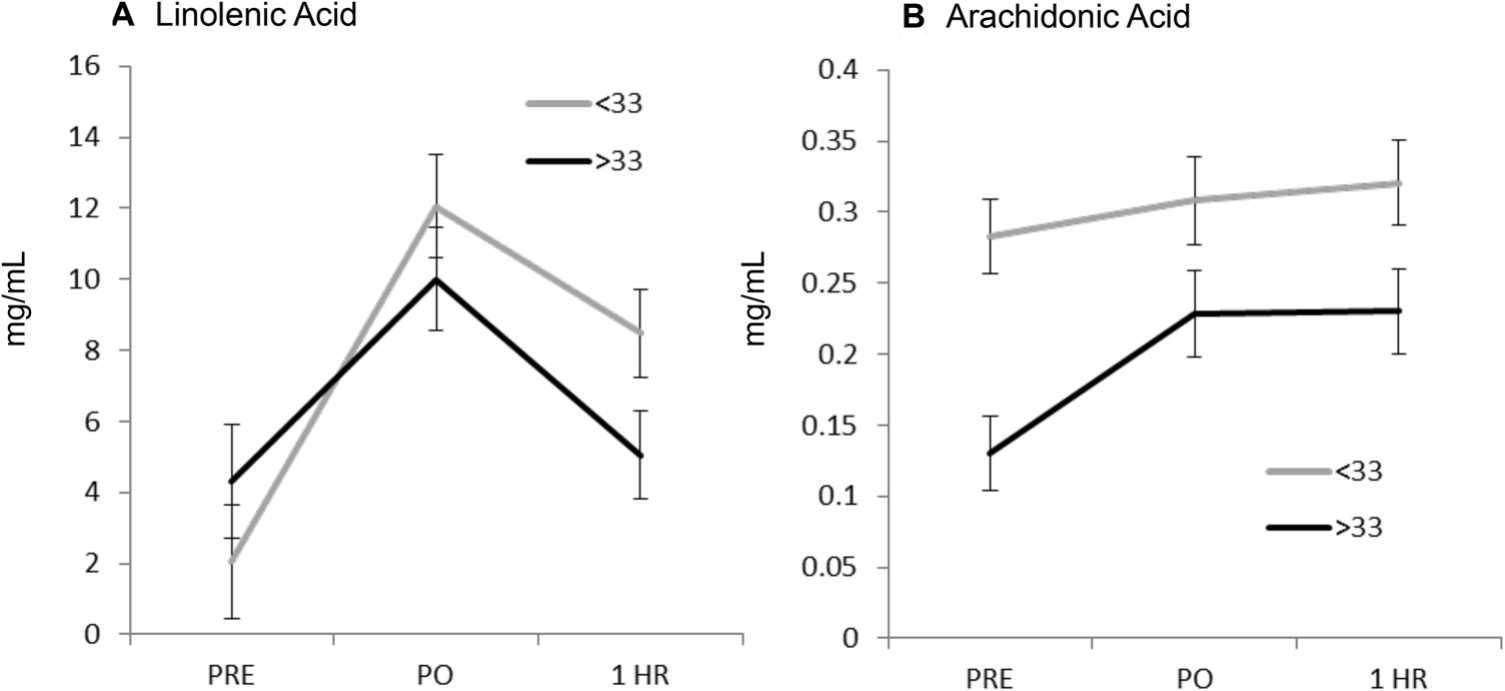
Linolenic and Arachidonic acid concentrations in the in plasma pre- and post-exercise in participants according to vitamin D status. The concentrations of linolenic (LA) and arachidonic acid (AA) were accurately determined using targeted analyses involving comparison with standard curves of LA and AA standards. The categories were sampled pre-exercise (PRE), immediately post-exercise (PO) and 1 h post-exercise (1HR). Participants with deficient (< 33 nmol/L) or non-deficient plasma vitamin D concentrations are represented by grey and black lines respectively

Linoleic acid (LA) was demonstrated to be one of the major sources of variation in the multivariate analysis. LA is a precursor to arachidonic acid (AA). AA formed eicosanoids function with diverse physiological and pathological roles in such inflammation or other immune responses. Therefore, the exact concentrations of both LA and AA were calculated in the samples (Fig. 4). These data confirmed increases in LA at 1 h post-exercise in the deficient vitamin D group (Fig. 4A) but AA appeared to be elevated at all exercise stages. Pearson’s correlation analysis showed a strong positive relationship between linoleic (LA) and arachidonic acid (AA) (correlation coefficient of 0.821; p = 0.000). In addition, the linear regression between the variables returned a significant association (R^2^ = 0.673, p < 0.001).

### Mucosal responses

There were no significant main effects of group, time or group × time interaction for saliva flow rate. Blood contamination was detected in the saliva samples from eight participants, leaving n = 5 and n = 10 in deficient and non-deficient groups respectively for sLac and sLys responses. Hence, effect sizes are also reported to support interpretation (Supplementary Information Table S1). There were no significant main effects of group or group × time interaction for salivary antimicrobial peptides when expressed as absolute concentration, secretion rate or relative to saliva osmolality (Supplementary Information Table S1). A main effect of time was revealed for sLac:osmolality (p = 0.014) but not sLac concentration (p = 0.103) or secretion rate (p = 0.416) (Supplementary Information Table S1). Post hoc analysis identified lower sLac:osmolality at post-exercise compared to pre-exercise (p = 0.012). There was no main effect of time for sLys concentration (p = 0.649), sLys secretion rate (p = 0.546) or sLys:osmolality (p = 0.208) (Supporting Information Table S1).

## Discussion

The aims of the study were to assess the impact of vitamin D status on immune and metabolomic responses to an acute bout of strenuous prolonged endurance exercise. To our knowledge, this is the first study to show that low plasma 25(OH)D is associated with greater decline in bacterial-stimulated degranulation in blood neutrophils following prolonged endurance exercise. This was coupled with novel findings of modulations in metabolomic signatures during the post-exercise period, whereby vitamin D status influenced exercise-induced changes in purine/pyrimidine as well as linoleic acid metabolism.

Athletes with deficient plasma 25(OH)D have previously been reported as having blunted monocyte-derived cytokines in antigen-stimulated whole blood culture [2]. Here, we suggest that aberrant responses to TLR agonists (both gram-positive and gram-negative bacteria) with deficient vitamin D status also occur with the major effector cell of innate immunity in response to acute strenuous endurance exercise. Compared to other innate phagocytes (e.g. macrophages), neutrophils appear to lack the intracellular 1α-hydroxylase enzyme that converts 25(OH)D to the biologically active 1,25-dihydroxyvitamin following activation of TLR signaling cascades by pathogenic antigens [36]. Release of antimicrobial activity in neutrophils can, however, be directly affected by 1,25(OH)_2_D alone through interaction with VDR or in synergy with TLR agonists [5]. Upon activation, the neutrophil releases a diverse array of granule-derived antimicrobial peptides (e.g. cathelicidin) and enzymes (neutrophil elastase). He et al. [2] and others have previously reported a positive correlation between plasma cathelicidin concentration and plasma 25(OH)D. In addition to being released upon degranulation of neutrophils and having direct antimicrobial effects, presence of such peptides are also known to have modulatory effects on neutrophil responses to bacterial stimulation [37, 38]. Although the exact effects may be direct and/or indirect, the available evidence does at least support the importance of vitamin D status in release of antimicrobial products from blood neutrophils.

A novel finding of the present study was the influence of vitamin D status on metabolomic responses to prolonged endurance exercise. The use of untargeted metabolomics as a system wide view of metabolic responses in exercise science research is receiving increasing attention [20, 23, 39–42]. Previous studies have shown a systematic shift in blood metabolites following strenuous exercise including heightened utilization of fuel substrates in several metabolic pathways, including increased glycolysis, lipolysis, adenine nucleotide catabolism, and amino acid catabolism [20, 22, 23]. Consistent with Nieman et al. [20, 23], we show that linoleic acid metabolism is a major source of variation in post-exercise metabolomic profiles. We, however, for the first time demonstrate circulating 25(OH)D concentrations may be important in mediating the exercise-induced changes in these pathways.

In a previous model of acute inflammatory stress (infection) that is likened to some of the responses to exercise [43], vitamin D was shown to stabilise metabolic changes [44]. Compared to a placebo group, vitamin D supplementation blunted increases in tricarboxylic acid intermediates, which were suggested to be accumulating as damage-associated molecular pattern molecules in response to stress [44]. A major metabolite contributing to the effect of vitamin D status in our study was linoleic acid, a polynunsaturated fatty acid that via action of oxidation or non-enzyamtic pathways leads to wide range of derivatives [45]. A role for post-exercise changes in oxidised linoleic acid metabolites (plasma 9 + 13 hydroxy-octadecadieonic acid (HODE)) in regulating innate immunity has previously been proposed following observed relationships with cytokine (IL-6, GCSF) concentrations known to regulate neutrophil function [41]. In our study, unlike linoleic acid, one of its derivatives arachidonic acid was significantly greater across all time points in those with low plasma (25OH)D. This is in line with previous metabolomic profiling studies where low vitamin D status has been associated with alterations in derivatives of linoleic acid (e.g. 9 + 13 HODE) [23], which are purported to be indicative of oxidative stress and inflammation [41]. Arachidonic acid is released from membrane phospholipases in response to non-specific activating stimuli (e.g. cytokines, hormones, stress) [46]. Although known to have direct effects, most of the biological role of arachidonic acid is attributable to its conversion by oxygenases (cyclooxygenase, lipoxygenase and P450) to eicosanoids (prostaglandins, leukotrienes) and other bioactive products that are effectors in immunity and in inflammation [46]. The hormonally active form of vitamin D, 25(OH)2D3, has been shown to regulate the expression of these inflammatory enzymes [47]. Further studies of the influence of vitamin D status on events upstream (e.g. phopsolipase A2 activity) and downstream of arachidonic acid (e.g. eicosanoid-derived molecules) using metabolomics and targeted lipidomic analyses will be important to understand how these metabolic disturbances relate to observed dysregulations in innate immunity of athletic populations.

Blunted blood neutrophil responses to in vitro stimuli are one of the classical measures of the “open window” period of immune dysfunction following acute prolonged endurance exercise. Unlike bacterial-stimulated neutrophil degranulation, we did not identify significant effects of vitamin D status on PMA-stimulated neutrophil oxidative burst. This may provide further evidence of the selective effect of vitamin D metabolites on receptor-mediated (TLR2/TLR4) intracellular activation cascades as opposed to the direct (receptor independent), and often labeled artificial stimulation of neutrophil responses via PMA [48]. The variance in exercise-induced changes innate immunity is considered to be best explained by exercise intensity [49]. Despite exposure to the same relative exercise workload and degree of physiological stress, as indicated by typical measures of HR, RPE, ${\dot{\text{V}}\text{O}}_{2}$, blood glucose, total and differential leukocyte counts, we observed differences in effector functions of innate immunity according to plasma 25(OH)D. Major sources of variation in the effect of vitamin D status on metabolomic responses to prolonged exercise did, however, include pyruvate metabolism (lactate) and tyrosine metabolism (adrenaline). It is possible that this global snapshot of metabolism indicates that participants with low plasma 25(OH)D experienced a greater physical stress response, and this may be a contributing factor in the immune responses. The previously reported associations of plasma 25(OH)D with performance of endurance exercise may be lend some support to this [50].

It has been suggested that plasma 25 (OH)D does not alter absolute numbers of circulating leukocytes but may modify proportions of lymphocyte subsets [16]. In the present study, we observed lower total lymphocytes and a greater neutrophil–lymphocyte ratio across all timepoints in those with low plasma 25(OH)D. Some have previously shown associations between plasma 25(OH)D and neutrophil:lymphocyte ratio [51]. Neutrophil:lymphocyte ratio is a marker of sympathetic nervous activation in asymptomatic healthy populations [14] and is increasingly considered to have a prognostic role in the clinical setting as a marker of inflammation (which corroborates with our metabolomic analysis) [52]. As there were no apparent effects on exercise-induced leukocyte trafficking according to vitamin D status, further evaluation, including underlying mechanisms and the clinical significance of differences in baseline leukocyte counts is required in athletic populations.

As mucosal membranes (e.g. oral cavity) are continuously exposed to potential pathogenic organisms, assessing immune markers in saliva, particularly AMPs has been of interest to understand exercise-induced changes in immunosurveillance [14]. A possible role for vitamin D in regulation of mucosal immunity has been proposed following numerous animal studies demonstrating the presence of VDR in the parotid, submandibular and sublingual salivary glands [16]. To our knowledge, this is the first study to investigate the influence of vitamin D status on mucosal responses following pronged endurance exercise. We did not observe any significant effects of vitamin D status on salivary AMPs. It must, however, be noted that the failure to demonstrate statistically significant exercise-induced changes between groups may be partly due to the loss of samples/participant numbers (as a result of blood contamination) for the salivary AMP measures. Vitamin D supplementation has been shown to increase resting salivary AMPs (cathelicidin) secretion rates [17], which was largely explained by changes in salivary flow rates over time. This corroborates with findings in animal studies where vitamin D_3_ treatment stimulated salivary flow rates via a calcium-dependent mechanism [53, 54]. Although the non-significant differences in temporal pattern of salivary flow rates between groups in this study may provide some evidence to support such modulation, the changes in absolute concentrations (e.g. lysozyme) may indicate that factors (e.g. expression by oral neutrophils and epithelial cells) other than salivary flow warrant further investigation for the influence of vitamin D on mucosal immunity.

When interpreting the results of this study, it is important to note the focus of our study was to examine the impact of low vitamin D status on responses to acute prolonged endurance exercise and not to determine optimal 25(OH)D concentrations. Defining thresholds for circulating vitamin D status remains a hotly debated topic whereby no precise thresholds for athletic populations exist particularly for immune health [16]. A common criticism is that studies state different classification values that lack scientific support and make comparison between studies difficult [55]. We classified our low vitamin D status on concentrations that have been shown to affect surrogate markers of innate immunity within previous studies ([2] < 30–33 nmol/l). Our study population was based on a secondary analysis of participants taking part in control arms of two randomised controlled trials. A larger, and appropriately powered, sample size is required to fully investigate the effects of a wide range of circulating 25(OH)D thresholds (i.e. different classifications of vitamin D status) on responses to acute endurance exercise. The positive correlation between plasma 25(OH)D and change in bacterial-stimulated neutrophil degranulation across our study population suggests that studies determining if there is in fact an optimum 25(OH)D concentration to maintain immunity are worthwhile. It would also be prudent for future studies to utilise a research design that will allow cause and effect to be established, including whether modifications of low circulating 25 (OH)D (e.g. by supplementation) attenuates impairment in innate immunity. We recognise that further investigation of immune regulation events at the level of TLR receptors and other aspects of neutrophil activation would further help understand and interpret the significance of the impact of vitamin D status.

In conclusion, we have shown for the first time that vitamin D status may be an important determinant of innate immune responses to a single bout of prolonged strenuous endurance exercise. These findings may have important implications on future between-group investigations of the effects of exercise and nutrition interventions on immunity and inflammation whereby the influence of vitamin D status may need to be accounted for within the study design or analysis. Our findings further establish the negative effects of low vitamin status on immune health in athletic populations and supports current advice of monitoring circulating 25(OH)D concentrations and the consideration of sensible sun exposure in summer months or a suitable vitamin D_3_ supplementation strategy in the winter months. Future research should assess the longer-term impact (e.g. incidence of URI) of vitamin D status on the variance in the magnitude of change in immunity following each bout of strenuous exercise. Such studies, in conjunction with those investigating the training-induced alterations in resting immunity will further strengthen the rationale for vitamin D supplementation to maintain immunity and resistance against pathogens.

## Supplementary Information

Below is the link to the electronic supplementary material.

Supplementary file1 (DOCX 20 KB)

## Data Availability

Data are available from the corresponding author upon reasonable request.
