# Supplementary material for: Vitamin D status modulates innate immune responses and metabolomic profiles following acute prolonged cycling
Source: Eur J Nutr. 2023 Jul 17;62(7):2977–90. doi: 10.1007/s00394-023-03181-1 (PMC10468936; doi:10.1007/s00394-023-03181-1)
Supplement: Supplementary file 1 — Supplementary file1 (DOCX 20 KB) [file 394_2023_3181_MOESM1_ESM.docx]

**Supplementary Information**

|  |  | |  | |  | | **p value** | | | | **partial eta squared** | | |
| --- | --- | --- | --- | --- | --- | --- | --- | --- | --- | --- | --- | --- | --- |
| **Immune measure** | **Pre-exercise** | | **Post-exercise** | | **1 h post-exercise** | | **group** | | **time** | **interaction** | **group** | **time** | **interaction** |
| **Saliva flow rate (mL⋅min^-1^)** | | | |  | |  | 0.224 | | 0.267 | 0.381 | 0.070 | 0.061 | 0.045 |
| Deficient | | 0.72 ± 0.32 | | 0.60 ± 0.26 | | 0.59 ± 0.22 |  | |  |  |  |  |  |
| Non-deficient | | 0.49 ± 0.31 | | 0.47 ± 0.31 | | 0.49 ± 0.26 |  | |  |  |  |  |  |
| **sLac concentration (mg⋅L^-1^ )** | | | |  | |  | 0.976 | | 0.103 | 0.372 | 0.00007 | 0.160 | 0.073 |
| Deficient | 4.6 ± 1.4 | | 4.8 ± 0.7 | | 4.8 ± 0.5 | |  | |  |  |  |  |  |
| Non-deficient | 4.3 ± 1.1 | | 5.2 ± 1.0 | | 5.1 ± 1.0 | |  | |  |  |  |  |  |
| **sLac secretion rate (μg⋅min^-1^ )** | | | |  | |  | 0.437 | | 0.416 | 0.371 | 0.047 | 0.065 | 0.073 |
| Deficient | 2.8 ± 1.0 | | 2.5 ± 0.8 | | 2.0 ± 0.4 | |  | |  |  |  |  |  |
| Non-deficient | 3.0 ± 2.0 | | 3.0 ± 1.9 | | 3.1 ± 1.4 | |  | |  |  |  |  |  |
| **sLac:osmolality (mg⋅mosmol^-1^ )** | | | |  | |  | 0.458 | | 0.014* | 0.481 | 0.047 | 0.297 | 0.059 |
| Deficient | 0.06 ± 0.02 | | 0.05 ± 0.01 | | 0.05 ± 0.01 | |  | |  |  |  |  |  |
| Non-deficient | 0.06 ± 0.02 | | 0.06 ± 0.02 | | 0.06 ± 0.02 | |  | |  |  |  |  |  |
| **sLys concentration (mg⋅L^-1^ )** | | | |  | |  | 0.521 | | 0.649 | 0.760 | 0.032 | 0.023 | 0.013 |
| Deficient | 29.0 ± 18.9 | | 28.2 ± 21.8 | | 19.7 ± 12.1 | |  | |  |  |  |  |  |
| Non-deficient | 18.9 ± 11.6 | | 18.7 ± 12.2 | | 19.8 ± 13.9 | |  | |  |  |  |  |  |
| **sLys secretion rate (μg⋅min^-1^ )** | | | |  | |  | 0.822 | | 0.546 | 0.518 | 0.004 | 0.049 | 0.046 |
| Deficient | 15.7 ± 12.6 | | 13.7 ± 12.2 | | 9.2 ± 5.3 | |  | |  |  |  |  |  |
| Non-deficient | 11.4 ± 7.8 | | 12.6 ± 12.0 | | 13.2 ± 10.0 | |  | |  |  |  |  |  |
| **sLys:osmolality (mg⋅mosmol^-1^)** | | | |  | |  | 0.953 | | 0.208 | 0.733 | 0.0003 | 0.127 | 0.016 |
| Deficient | 0.34 ± 0.28 | | 0.26 ± 0.28 | | 0.27 ± 0.17 | |  |  | |  |  |  |  |
| Non-deficient | 0.31 ± 0.19 | | 0.26 ± 0.15 | | 0.28 ± 0.17 | |  |  | |  |  |  |  |

Table S1. Salivary flow rate and antimicrobial peptides responses according to plasma 25(OH)D concentration.

Significant main effect of time (*p < 0.05)
